# Triosephosphate isomerase of *Streptococcus pneumoniae* is released extracellularly by autolysis and binds to host plasminogen to promote its activation

**DOI:** 10.1002/2211-5463.13396

**Published:** 2022-03-29

**Authors:** Satoru Hirayama, Hisanori Domon, Takumi Hiyoshi, Toshihito Isono, Hikaru Tamura, Karin Sasagawa, Fumio Takizawa, Yutaka Terao

**Affiliations:** ^1^ Division of Microbiology and Infectious Diseases Niigata University Graduate School of Medical and Dental Sciences Japan; ^2^ Center for Advanced Oral Science Niigata University Graduate School of Medical and Dental Sciences Japan; ^3^ Division of Periodontology Niigata University Graduate School of Medical and Dental Sciences Japan

**Keywords:** autolysin, plasmin, plasminogen, *Streptococcus pneumoniae*, triosephosphate isomerase, virulence strategy

## Abstract

Recruitment of plasminogen is an important infection strategy of the human pathogen *Streptococcus pneumoniae* to invade host tissues. In *Streptococcus aureus*, triosephosphate isomerase (TPI) has been reported to bind plasminogen. In this study, the TPI of *S. pneumoniae* (TpiA) was identified through proteomic analysis of bronchoalveolar lavage fluid from a murine pneumococcal pneumonia model. The binding kinetics of recombinant pneumococcal TpiA with plasminogen were characterized using surface plasmon resonance (SPR, Biacore), ligand blot analyses, and enzyme‐linked immunosorbent assay. Enhanced plasminogen activation and subsequent degradation by plasmin were also shown. Release of TpiA into the culture medium was observed to be dependent on autolysin. These findings suggest that *S*. *pneumoniae* releases TpiA via autolysis, which then binds to plasminogen and promotes its activation, thereby contributing to tissue invasion via degradation of the extracellular matrix.

## Graphical abstract

Triosephosphate isomerase (TpiA) of *Streptococcus pneumoniae* is a glycolytic enzyme that functions within the bacterial cell but is released extracellularly by autolysis and localizes to the bacterial cell surface. TpiA binds to host plasminogen and promotes its activation. TpiA functions to recruit host plasminogen and promote its conversion to plasmin, suggesting that it contributes to tissue invasion, including matrix degradation.

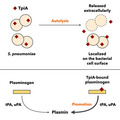

**Abbreviations**
A_405_
: absorbance at 405 nm
ANOVA: analysis of variance
BALF: bronchoalveolar lavage fluid
CBB: coomassie brilliant blue
EACA: ε‐aminocaproic acid
ECM: extracellular matrix
FDR: false discovery rate
GAPDH: glyceraldehyde‐3‐phosphate dehydrogenase
HCD: higher‐energy collisional dissociation
HEPES: 4‐(2‐hydroxyethyl)‐1‐piperazineethanesulfonic acid
HRP: horseradish peroxidase
iTRAQ: isobaric tags for relative and absolute quantification
KEGG: Kyoto Encyclopedia of Genes and Genomes
LC: liquid chromatography
LPxTG: leucine, proline, any amino acid, threonine, and glycine
MS: mass spectrometry
NCBI: National Center for Biotechnology Information
OD_600_
: optical density at 600 nm
PBST: PBS supplemented with 0.05% Tween 20
PSM: peptide‐spectrum match
PVDF: polyvinylidene fluoride
PepO: endopeptidase O
rTpiA: recombinant TpiA
SPR: surface plasmon resonance
TBST: tris‐buffered saline containing 0.05% Tween 20
TCA: trichloroacetic acid
THY: Todd‐Hewitt broth supplemented with 0.5% yeast extract
tPA: tissue‐type plasminogen activator
TPI: triosephosphate isomerase
Tuf: elongation factor Tu
uPA: urokinase‐type plasminogen activator
Δ*lytA*
: *lytA*‐negative mutant

Infections caused by *Streptococcus pneumoniae* are prevalent worldwide, with particularly high morbidity and mortality in children and the elderly [1]. In 2005, the World Health Organization estimated that 1.6 million deaths per year were caused by *S. pneumoniae* [1]. *S. pneumoniae* is a commensal bacterium that settles in the human nasopharynx, causing not only noninvasive diseases such as otitis media, sinusitis, and pneumonia, but also invasive and life‐threatening diseases such as bacteremia, sepsis, and meningitis.

For pathogenic bacteria to invade host tissues and the bloodstream, proteolysis of the extracellular matrix (ECM) of host cells is required. *S. pneumoniae* recruits plasminogen to the bacterial cell surface to degrade ECM. Plasminogen bound to the pneumococcal cell surface is activated to plasmin by tissue‐type plasminogen activator (tPA) and/or urokinase‐type plasminogen activator (uPA), which exhibits proteolytic activity [2, 3]. Several pneumococcal proteins, such as alpha‐enolase [4, 5, 6, 7, 8, 9, 10], have been previously reported to bind plasminogen.

In *Staphylococcus aureus*, triosephosphate isomerase (TPI) has been reported to bind plasminogen [11]. TPI is an enzyme that catalyzes the fifth step of the glycolytic system, reversibly isomerizing glyceraldehyde‐3‐phosphate and dihydroxyacetone phosphate [12], and is present in all organisms [12, 13, 14]. In general, the molecular weight of the monomer chain of TPI is approximately 27 000, and TPI is active only in dimers [12]. Thus, in *S*. *aureus*, TPI is involved in energy production intracellularly, but extracellularly, it binds to plasminogen and functions as a so‐called moonlighting protein.

Here, we collected bronchoalveolar lavage fluid (BALF) from a murine pneumococcal pneumonia model and performed proteomic analysis to identify pneumococcal proteins from an *in vivo* sample. Among the identified proteins was TpiA, the TPI of *S. pneumoniae*. In this study, we analyzed the interaction between TpiA and plasminogen to investigate its involvement in infection.

## Materials and methods

### Bacterial strains and growth conditions

Wild‐type and *lytA*‐negative mutant (Δ*lytA*) [15] strains of *S. pneumoniae* D39 were used. These pneumococcal strains were cultured in THY (Todd‐Hewitt broth [Becton, Dickinson, Franklin Lakes, NJ, USA] supplemented with 0.5% yeast extract) broth at 37 °C. Precultured seeds (optical density at 600 nm [OD_600_] of 0.1) were inoculated at a dilution of 1 : 200 into fresh THY broth and incubated at 37 °C, and growth curves were generated by sequential measurement of OD_600_ with a spectrophotometer miniphoto 518R (TAITEC, Saitama, Japan).

*Brevibacillus choshinensis* HPD31‐SP3 was used to construct rTpiA. Transformant of this strain was cultured on MTNm plate (10 g·L^−1^ glucose, 10 g·L^−1^ phytone peptone, 5.75 g·L^−1^ ehrlich bonito extract [35%], 2 g·L^−1^ yeast extract, 10 mg·L^−1^ FeSO_4_•7H_2_O, 10 mg·L^−1^ MnSO_4_•4H_2_O and 1 mg·L^−1^ ZnSO_4_•7H_2_O, adjusted pH 7.0, and supplemented with 15 g·L^−1^ agar, 20 mm MgCl_2_, and 50 µg·mL^−1^ neomycin) or in TMNm (MTNm without agar and MgCl_2_) or 2SYNm (20 g·L^−1^ glucose, 40 g·L^−1^ Bacto soytone, 5 g·L^−1^ Bacto yeast extract, 0.15 g·L^−1^ CaCl_2_•2H_2_O, adjusted pH 7.2, and supplemented with 50 µg·mL^−1^ neomycin) broths.

### Construction of the *Brevibacillus* strain producing rTpiA

The rTpiA of *S. pneumoniae* D39 was constructed using the *Brevibacillus in vivo* cloning system (Takara Bio, Shiga, Japan). The open reading frame of the *tpiA* gene, excluding the start codon, was amplified from *S. pneumoniae* D39 genomic DNA using primers with sequences homologous to plasmid pBIC2 (Table 1, pBIC‐tpiA‐F and pBIC‐tpiA‐R). Polymerase chain reaction (PCR) was performed using the DNA polymerase KOD‐Plus‐Ver. 2 (Toyobo, Osaka, Japan), according to the manufacturer’s instructions. The amplified DNA fragments were confirmed by agarose gel electrophoresis and then purified using a QIAquick PCR Purification Kit (QIAGEN, Hilden, Germany). pBIC2 (50 ng) and purified DNA fragments were mixed at a molar ratio of 1 : 2 and transformed using *B*. *choshinensis* HPD31‐SP3 competent cells according to the manufacturer’s instructions. Transformed clones were selected on an MTNm plate. Some transformants were inoculated into 2SYNm broth and incubated overnight at 37 °C with shaking at 120 r.p.m. The bacteria were collected from the culture media, and the plasmids were extracted using a QIAprep Spin Miniprep Kit (QIAGEN). Sequencing of the DNA inserted into the plasmid was entrusted to Eurofins Genomics (Tokyo, Japan) using the primers for sequencing (Table 1, pBIC‐ins‐F and pBIC‐ins‐R), and clones with the correct sequence were selected.

**Table 1:** The primers used in this study.

Name	Sequence (5’‐3’)
pBIC‐tpiA‐F	GATGACGATGACAAATCACGTAAACCATTTATCGCTG
pBIC‐tpiA‐R	CATCCTGTTAAGCTTGCTACTTACTGATTATTTTACAAAGTCAAGC
pBIC‐ins‐F	CGCGATATCAGGATTCGG
pBIC‐ins‐R	CAATGTAATTGTTCCCTACCTGC

### Purification of rTpiA

*B. choshinensis* HPD31‐SP3‐expressing rTpiA was cultured in TMNm broth at 32 °C for 64 h with shaking at 120 r.p.m. The culture supernatant was collected by centrifugation (12 000 × ***g*** for 20 min) at 4 °C and filter‐sterilized through a 0.45‐µm polyvinylidene fluoride (PVDF) filter (Merck Millipore, Darmstadt, Germany). Ni Sepharose 6 Fast Flow (Cytiva, Marlborough, MA, USA) was resuspended in binding buffer (20 mm NaHPO_4_, pH 7.4, 0.5 m NaCl, and 40 mm imidazole) to make a 50% slurry. The slurry (1 mL) was added to a chromatography column (Bio‐Rad, Hercules, CA, USA), and the flow‐through was discarded. NaCl and imidazole were added to the supernatant so that the final concentrations were 0.5 m and 40 mm, respectively, and the sample (~ 10 mL) was applied to the column with a stopper. The column was incubated with the lid in place for 1 h and then rotated at room temperature. After discarding the flow‐through, the column was washed four times with binding buffer. Proteins trapped in the column by His‐tag and Ni interaction were extracted four times with 500 μL of elution buffer (20 mm NaHPO_4_, pH 7.4, 0.5 m NaCl, and 500 mm imidazole). If necessary, the extracted samples were desalted and the solvent was replaced with PBS using PD‐10 columns (GE Healthcare, Chicago, IL, USA).

### Animals

Male C57BL/6J mice (8‐week‐old) were purchased from Charles River Laboratories (Kanagawa, Japan). Mice were maintained in ventilated cages and provided with sterile food and water ad libitum under specific pathogen‐free conditions, and acclimatized by keeping them in this environment for 1 week.

### Murine pneumococcal pneumonia model and BALF sampling

A mouse was infected with *S. pneumoniae*, as previously described [16], with some modifications. Briefly, a 9‐week‐old mouse was anesthetized with a mixture of medetomidine hydrochloride, midazolam, and butorphanol and intratracheally infected with *S. pneumoniae* D39 wild‐type strain (1.0 × 10^9^ cells in 50 μL PBS) using a MicroSprayer Aerosolizer (Penn‐Century Inc., Philadelphia, PA, USA). An uninfected mouse received only 50 µL of PBS. BALF sampling was performed as described previously [17]. Mice were slaughtered 24 h after infection, and BALF was sampled by injecting 1 mL of PBS through the bronchus using a 19G needle and slowly aspirating. The proteomic analysis of BALF using isobaric tags for relative and absolute quantification (iTRAQ) [18] was subcontracted to APRO Science (Tokushima, Japan).

### iTRAQ analysis

To concentrate the protein, 20% trichloroacetic acid (TCA) was added to each sample to achieve a final concentration of 10%. The samples were vortexed, incubated on ice for 30 min, and centrifuged at 18 800 × ***g*** for 30 min at 4 °C. Ice‐cold acetone (500 µL) was then added to the resulting pellet, and the mixture was vortexed. The mixture was then centrifuged (18 800 × ***g*** for 30 min) at 4 °C. The resulting pellet was washed twice with 500 µL ice‐cold acetone and resuspended in iTRAQ lysis buffer (50 mm triethylammonium bicarbonate and 0.1% sodium dodecyl sulfate). The protein concentrations were determined using the bicinchoninic acid assay [19]. BALF samples were reduced, alkylated, and digested with trypsin (AB Sciex, Framingham, MA, USA) overnight at 37 °C, according to iTRAQ manufacturer's instructions. iTRAQ labeling was performed according to the manufacturer's instructions using an iTRAQ Reagent‐8 Plex Kit (AB Sciex). The labeled samples were combined and fractionated into six fractions using an ICAT strong cation exchange column (AB Sciex), following the manufacturer's instructions. Each fraction was concentrated using vacuum centrifugation and resuspended in 200 µL of 0.1% (v/v) formic acid. Samples were desalted using a MonoSpin C18 column (GL Science, Tokyo, Japan). The desalted samples were concentrated using vacuum centrifugation and resuspended in 40 µL of 0.1% (v/v) formic acid. All samples were stored at –20 °C until they could be analyzed via LC‐MS. Each peptide fraction was analyzed using Q Exactive Plus (Thermo Fisher Scientific, Waltham, MA, USA) coupled online with a capillary high performance liquid chromatography system (EASY‐nLC 1200, Thermo Fisher Scientific) to acquire the MS/MS spectra. One microgram per fraction was loaded onto a Thermo Scientific EASY‐Spray 75 µm × 15 cm column (3‐μm particle diameter, 100 Å pore size; Thermo Fisher Scientific) maintained at a flow rate of 300 nL·min^−1^. The sample was separated using a gradient of 0.1% formic acid in water (A) and 0.1% formic acid in 80% acetonitrile (B). The gradient conditions were as follows: 5.0% B initially, increased to 35% B in 100 min, followed by a final increase to 100% B in 2 min with an 8 min isocratic hold. The mass spectrometer was operated in the positive ion mode, and the normalized collision energy was 30%. The MS data were collected by dynamically selecting the top ten most expressed precursor ions in the survey scan (350–1500 m/z) for higher‐energy collisional dissociation (HCD) fragmentation. Survey scans were obtained at a resolution of 70 000 at m/z 200; the automatic gain control target was 1e6, and the maximum injection time was 60 ms. The HCD resolution spectra were 17 500 at m/z 200, the automatic gain control target value was 5e4, and MS was dynamically excluded for 60 s. Proteome Discoverer (version 2.2, Thermo Fisher Scientific) was used to search against the NCBI *S. pneumoniae* D39 database. The options used to identify proteins were as follows: peptide mass tolerance = 10 p.p.m., MS/MS tolerance = 0.02 Da, enzyme = trypsin, missed cleavage = 2, fixed modification: iTRAQ4plex (K), iTRAQ8plex (N‐term), variable modification: oxidation (M), Methylthio (C), iTRAQ4plex (Y), FDR ≦ 1% (PSM and protein level).

### SDS/PAGE, western blotting, and far‐western blotting

SDS/PAGE and western blotting were performed as previously described [20, 21], with some modifications. Protein samples (plasminogen from human plasma (Sigma‐Aldrich, Darmstadt, Germany), laminin from human placenta (Sigma‐Aldrich), (BSA, Sigma‐Aldrich), and rTpiA) prepared in PBS or water were separated by Tris‐glycine SDS/PAGE using 12.5% polyacrylamide gels (ATTO, Tokyo, Japan) and stained with Coomassie Brilliant Blue (CBB, Quick‐CBB; FUJIFILM Wako Pure Chemical, Osaka, Japan). Culture supernatants of *S. pneumoniae* D39 were filtered with 0.22‐µm PVDF filters (Merck Millipore) and concentrated 10‐fold by TCA precipitation, and then subjected to SDS/PAGE. For western blotting, gels were electroblotted onto PVDF membranes (Merck Millipore), followed by incubation with blocking reagent (5% BSA or 5% skim milk). The membranes were probed with rabbit polyclonal antibodies against plasminogen (GeneTex, Irvine, CA, USA), laminin (Sigma‐Aldrich), or TpiA (peptide antibody obtained by subcontracting to Eurofins Genomics, peptide sequence: NH_2_‐C+GTGKSASQDDAQKM‐COOH) in Tris‐buffered saline containing 0.05% Tween‐20 (TBST) and blocking reagent. Horseradish peroxidase (HRP)‐conjugated goat anti‐rabbit IgG (Cell Signaling Technology, Beverly, MA, USA) was used as the secondary antibody at a dilution of 1 : 3000. Following addition of the substrate (ECL Select western blotting Detection Reagent; Cytiva), chemiluminescence was visualized using a LAS‐4000 mini (Fujifilm, Tokyo, Japan).

To determine the binding of rTpiA and BSA to human plasminogen and laminin, far‐western blotting assays were performed according to a previously described method [22], with some modifications. rTpiA and BSA were subjected to SDS/PAGE, electroblotted onto PVDF membranes, and blocked in the same manner as described above. The membranes were washed with TBST and incubated for 90 min with 30 µg·mL^−1^ of human plasminogen or laminin in TBST containing blocking reagent at room temperature. Thereafter, antibodies recognizing plasminogen and laminin and secondary antibodies were used to probe and detect human proteins attached to rTpiA and BSA, as in the western blotting method described above.

### Estimation of binding activity of the proteins by surface plasmon resonance assay

The molecular basis of the binding of rTpiA to human plasminogen, laminin, and BSA was investigated using surface plasmon resonance (SPR)‐based binding techniques, as previously described [11, 23, 24], with some modifications. Briefly, SPR measurements were performed using a Biacore X100 instrument (GE Healthcare). rTpiA was diluted with 10 mm sodium acetate buffer (pH 4) to a concentration of 50 μg·mL^−1^ and immobilized on a Series S Sensor Chip CM5 (Cytiva) using an amine coupling kit according to the manufacturer’s instructions. Human plasminogen, laminin, and BSA were diluted with running buffer (100 mm HEPES pH 7.4, containing 150 mm NaCl, 3 mm EDTA, and 0.005% surfactant P20) and injected into the flow cell. The flow rate was maintained at 10 μL·min^−1^ for immobilization and at 20 μL·min^−1^ for analysis. Regeneration of the surface on the sensor chip was achieved by using 50 mm HCl. The data were analyzed using the Biacore X100 evaluation software (GE Healthcare).

### ELISA

To measure the binding of rTpiA to host proteins, we performed an ELISA based on a previously reported method [20], with some modifications. Host proteins (plasminogen, laminin, fibronectin from human plasma [Sigma‐Aldrich], elastin from human lung [Elastin Products Company, Owensville, MO, USA], and fibrinogen from human plasma [Sigma‐Aldrich]) and BSA were diluted in coating buffer (Na_2_CO_3_ 1.59 g·L^−1^, NaHCO_3_ 2.93 g·L^−1^, NaN_3_ 0.2 g·L^−1^ in water, and pH 9.6). These were dispensed into 96‐well Half Area Clear Flat Bottom Polystyrene High Bind Microplates (Corning, Corning, NY, USA) at 1 µg per well and incubated at 4 °C overnight. The liquid in the wells was discarded, and the wells were incubated with 1% skim milk in PBS supplemented with 0.05% Tween‐20 (PBST) at 37 °C for 2 h for blocking. The wells were washed with PBST thrice, 1 µg per well of rTpiA was then added, and the mixture was incubated at 37 °C for 1 h. To examine whether the lysine residues in rTpiA affect binding to the host proteins, we also performed an experiment in which ε‐aminocaproic acid (EACA, Sigma‐Aldrich) was added along with rTpiA. After washing the wells thrice with PBST, anti‐TpiA peptide antibody diluted 1 : 1000 in 0.5% skim milk in PBST was added and incubated at 37 °C for 1 h. After washing the wells thrice with PBST again, alkaline phosphatase‐linked anti‐rabbit IgG (H + L) (Bethyl Laboratories, Montgomery, TX, USA) diluted 1 : 5000 in 0.5% skim milk in PBST was added and incubated at 37 °C for 1 h. After washing thrice with PBST once more, 3 g·L^−1^ disodium *p*‐nitrophenylphosphate hexahydrate dissolved in diethanolamine buffer (diethanolamine 9.7 mL·L^−1^, NaN_3_ 0.2 g·L^−1^, MgCl_2_•6H_2_O 0.1 g·L^−1^ in water, pH 9.6) was added. The samples were incubated at 37 °C, and the absorbance at 405 nm (A_405_) was measured using a microplate spectrophotometer (Multiskan FC, Thermo Fisher Scientific).

### Plasminogen activation

To investigate the effect of rTpiA on plasminogen activation, plasminogen was activated to plasmin using tPA or uPA, according to a previously reported method [11] with some modifications. All reagents and proteins were prepared in PBS (pH 7.4). First, 2 μg of plasminogen (in 80 µL PBS) and 2.5 to 40 pmol of rTpiA or BSA (in 10 µL PBS) were mixed and incubated at 37 °C for 30 min in a 96‐well microtiter plate. Next, 10 µL of 10 µg·mL^−1^ tPA (Recombinant human tPA; NKMAX, Sungnam, Korea) or 10 µg·mL^−1^ uPA (Recombinant human uPA; NKMAX) and 100 µL of 0.45 mm S‐2251 substrate (DiaPharma Group, West Chester, OH, USA) were added to the wells, and incubated at 37 °C. The release of *p‐*nitroaniline upon degradation of S‐2251 by plasmin was monitored by sequentially measuring A_405_ using a microplate spectrophotometer (Multiskan FC).

### Interaction of rTpiA and plasmin

To investigate the interaction between rTpiA and plasmin, they were mixed according to a previously described method [11] with some modifications. rTpiA (2 µg) and 2 µg of plasmin (plasmin solution from human plasma; fujifilm Wako Pure Chemical) were mixed in 50 µL of PBS. After incubation at 37 °C for 3 h, the mixture was subjected to SDS/PAGE and western blotting, and detection was carried out using antibodies against plasminogen and TpiA as described above.

### Statistical analysis

Unless otherwise stated, data were statistically evaluated using two‐way ANOVA, and Sidak’s multiple comparisons test was used. All statistical analyses were performed using Prism 8 software version 8.4.3 (GraphPad Software, La Jolla, CA, USA). Statistical significance was set at *P* < 0.05.

### Study approval

The safety committee for genetic modification experiments at Niigata University approved the genetic recombination experiments (approval no. SD01332). All animal experiments were approved by the Institutional Animal Care and Use Committee of Niigata University (approval no. SA00451).

## Results

### TPI was identified in BALF from murine pneumococcal pneumonia model

To identify the *S*. *pneumoniae* proteins from an *in vivo* sample, we analyzed BALF from a mouse infected with *S. pneumoniae*. Although 1911 proteins of *S. pneumoniae* D39 are currently annotated in the Kyoto Encyclopedia of Genes and Genomes (KEGG) [25], only 15 proteins were identified in BALF samples, which was not even 1% (Table 2). Therefore, we hypothesized that these 15 proteins might include those involved in pneumococcal infection of mouse alveoli. Among these identified proteins, we focused on TPI (Table 2, no. 7), which has been shown to contribute to infection in other bacterial species, for further investigation.

**Table 2:** The pneumococcal proteins identified by iTRAQ analysis in BALF from a murine pneumococcal pneumonia model.

No.	Description	Accession	Coverage^a^	# Peptides^b^	# PSMs^c^	Molecular mass/kDa	Theoretical pI
1	Transcriptional regulator, putative	ABJ53817.1	42.19	1	1	7.53	4.84
2	Ribosomal protein L7/L12	ABJ54917.1	25.41	3	7	12.44	4.49
3	Phosphopyruvate hydratase	WP_000022815.1	21.66	6	15	47.07	4.81
4	Glyceraldehyde‐3‐phosphate dehydrogenase, type I	ABJ54738.1	20.90	6	13	35.83	5.47
5	50S ribosomal protein L29	Q04MM8.1	19.12	1	1	7.98	9.57
6	Elongation factor Tu	WP_001040724.1	16.33	4	6	43.94	4.97
7	Chain E, triosephosphate isomerase	5IBX_E	5.49	1	1	26.81	4.84
8	Fructose‐bisphosphate aldolase	WP_001019003.1	3.07	1	1	31.38	5.10
9	ATP synthase subunit beta	Q04HT9.1	2.99	1	1	50.87	4.94
10	Excinuclease ABC subunit UvrC	WP_001061169.1	2.77	1	1	70.41	8.76
11	ABC transporter, transmembrane protein Vexp3	ABJ53925.1	2.40	1	1	49.95	8.79
12	Fe‐S cluster assembly ATPase SufC	WP_000114489.1	2.34	1	1	28.39	4.72
13	NADP‐specific glutamate dehydrogenase	ABJ55261.1	1.79	1	1	48.77	5.55
14	KH domain protein	ABJ55165.1	1.49	1	1	60.19	5.41
15	ATP‐dependent Clp protease ATP‐binding subunit	WP_001109677.1	0.74	1	2	90.12	6.01

^a^ Coverage ratio calculated by dividing the number of amino acids in all peptides found by the number of amino acids in the entire protein sequence.

^b^ The number of different peptide sequences in a protein.

^c^ The total number of identified peptide sequences (peptide spectrum matches) for the protein.

### rTpiA binds to human plasminogen

**Fig. 1:**
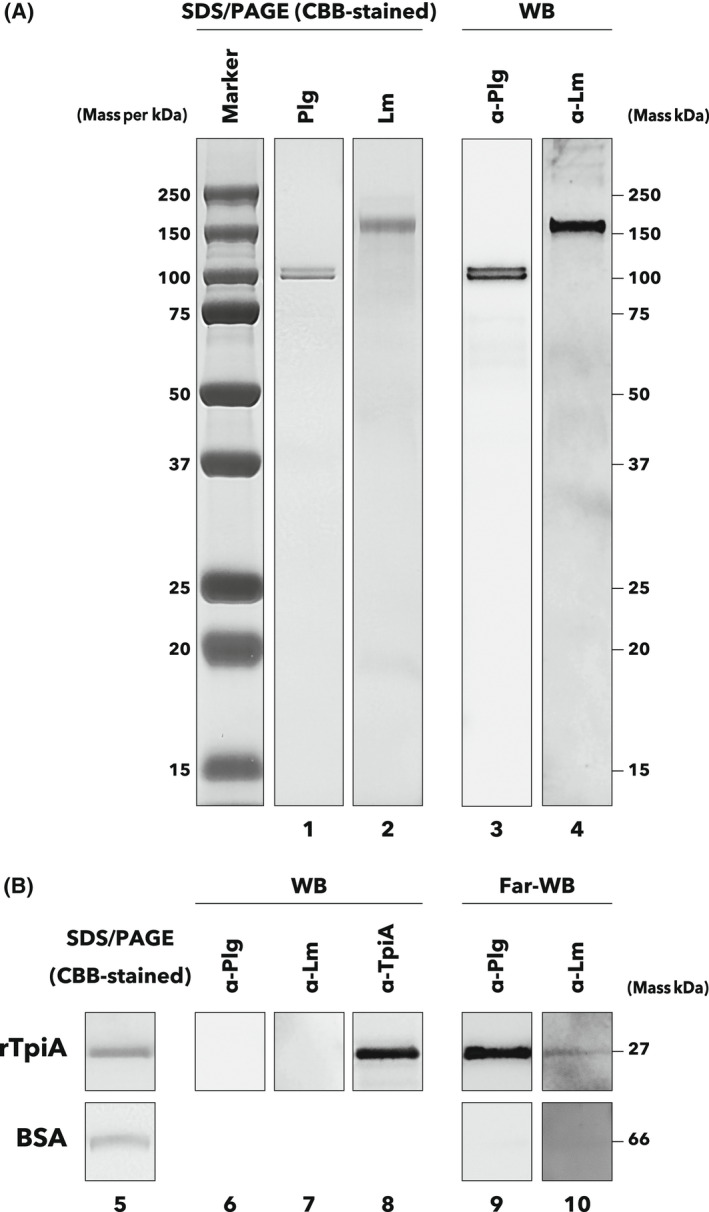
Binding of rTpiA to host proteins. (A) Electrophoretic analysis of human plasminogen (Plg) and laminin (Lm). Plasminogen (200 ng) and laminin (1 µg) were subjected to SDS/PAGE and stained with CBB (nos. 1 and 2). Precision Plus Protein Dual Color Standards (Bio‐Rad) were used as markers. For western blotting analysis, plasminogen (20 ng) and laminin (200 ng) were subjected to SDS/PAGE, and the proteins were electroblotted onto PVDF membranes. The membranes were incubated with primary antibodies of antiplasminogen (α‐Plg, 1 : 5000 dilution, no. 3) or antilaminin (α‐Lm, 1 : 3000 dilution, no. 4) and further incubated with HRP‐conjugated secondary antibody (1 : 3000 dilution). (B) Binding assay of rTpiA to plasminogen and laminin by far‐western blotting. rTpiA (200 ng) and BSA (200 ng) were subjected to SDS/PAGE, and the proteins were electroblotted onto PVDF membranes. The membranes were incubated with 30 µg·mL^−1^ of plasminogen or laminin, followed by incubation with antiplasminogen (1 : 5000 dilution, no. 9) or antilaminin (1 : 3000 dilution, no. 10) primary antibodies, and then with HRP‐conjugated secondary antibody (1 : 3000 dilution). SDS/PAGE and CBB staining were also performed for rTpiA and BSA (no. 5). Western blotting confirmed that rTpiA did not react with antiplasminogen and antilaminin antibodies but reacted with anti‐TpiA peptide antibody (α‐TpiA, 1 : 500 dilution) (nos. 6, 7, and 8). Detection of chemiluminescence due to enzymatic activity of HRP was performed with an exposure time of less than 10 s. These experiments were conducted at least thrice, and the same results were obtained. The original blot images are shown in Fig. S1.

We prepared rTpiA and analyzed its interaction with the host proteins human plasminogen and laminin. SDS/PAGE and western blotting indicated that antiplasminogen and antilaminin antibodies were specific for each protein (Fig. 1A). Next, the interaction of rTpiA with each protein was analyzed by far‐western blotting. Figure 1B shows that rTpiA has binding properties with both plasminogen and laminin (nos. 9 and 10). We confirmed that rTpiA did not react with antiplasminogen or antilaminin antibodies (Fig. 1B, no. 6‐8). On the other hand, BSA did not show any interaction with plasminogen or laminin (Fig. 1B, no. 9, 10).

**Fig. 2:**
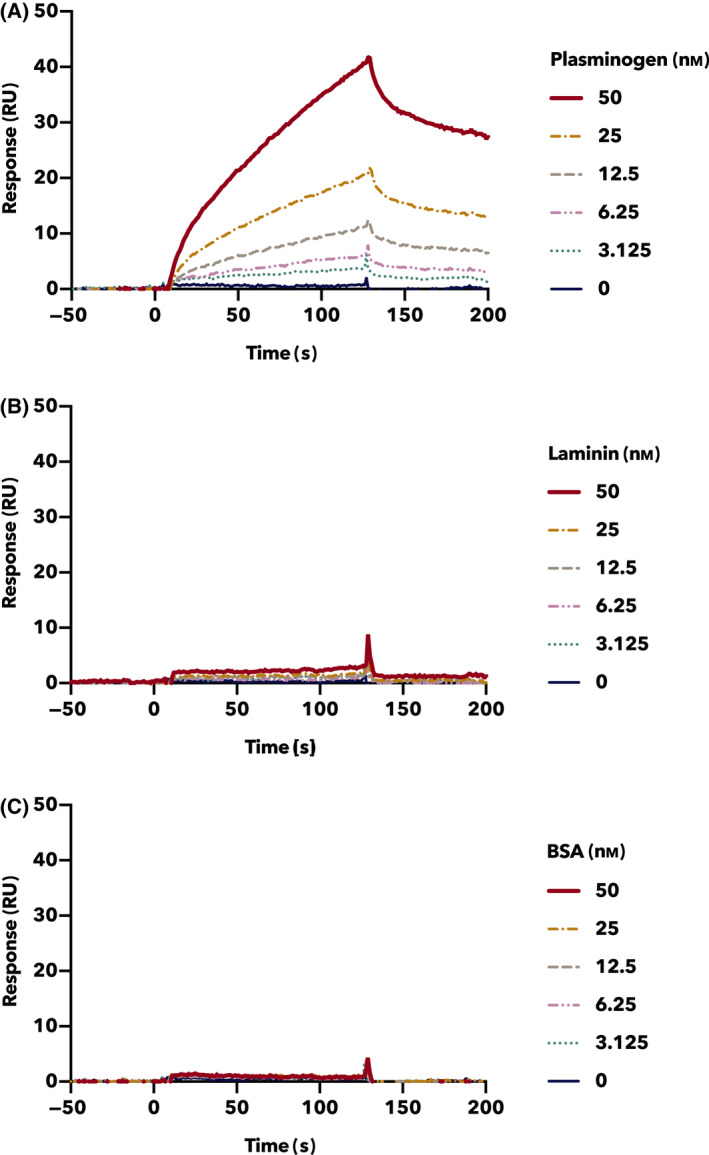
Binding activity of rTpiA to proteins by SPR. The binding of rTpiA to human plasminogen (A), laminin (B), and BSA (C) was analyzed by SPR. Plasminogen, laminin, and BSA were prepared and applied at 3.125 to 50 nm and reacted with rTpiA immobilized on the sensor chip with a contact time of 120 s. These experiments were conducted at least thrice, and similar results were obtained.

The binding profiles of rTpiA to each protein were analyzed using SPR and its application software. Consistent with the results shown in Fig. 1, human plasminogen clearly bound to the biosensor‐attached rTpiA in a dose‐dependent manner (Fig. 2A). The apparent association rate (*k_a_*), dissociation rate (*k_d_*), and dissociation (K_D_) of rTpiA and plasminogen were *k_a_* = 1.34 × 10^4^ 1/Ms, *k_d_* = 5.53 × 10^−3^ 1/s, and K_D_ = 4.11 × 10^−7^ m, respectively. The *k*_a_, *k*_d_, and K_D_ of both laminin and BSA were not calculated against rTpiA because both laminin and BSA demonstrated almost no binding to rTpiA at the same dose as plasminogen (Fig. 2B,C).

### Binding of rTpiA to plasminogen depending on the lysine residues of rTpiA

**Fig. 3:**
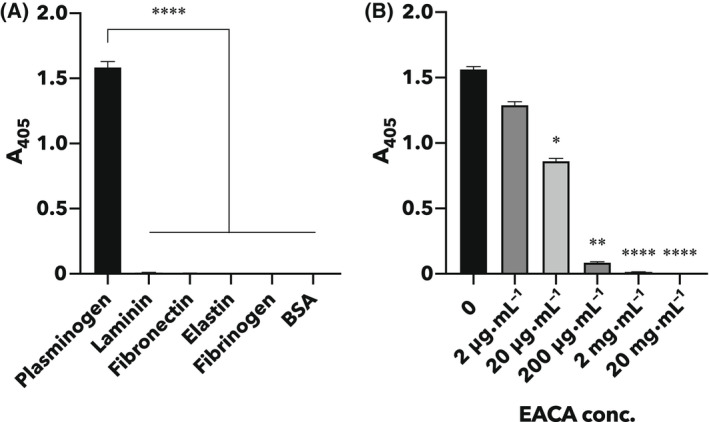
Binding of rTpiA to plasminogen is inhibited by a lysine analog. (A) ELISA results that measured the binding of rTpiA to each host protein (plasminogen, laminin, fibronectin, elastin, and fibrinogen) and BSA. After incubation with rTpiA in the wells of each protein‐coated microtiter plate, the bound rTpiA was detected using the anti‐TpiA peptide antibody and AP‐conjugated secondary antibody. Error bars indicate standard error (*n* = 7). Asterisks indicate significant differences between groups (****P < 0.0001, Tukey’s multiple comparisons test). (B) ELISA results obtained by adding EACA along with rTpiA to the wells of a plasminogen‐coated microtiter plate. Error bars indicate standard error (*n* = 6). Asterisks indicate significant differences between groups (*P < 0.05, **P < 0.01, ****P < 0.0001, Dunn’s multiple comparisons test). All results were measured at 15 min after substrate addition.

The binding of rTpiA to various host proteins (plasminogen, laminin, fibronectin, elastin, and fibrinogen) and BSA was measured using ELISA, which showed significant binding only to plasminogen (Fig. 3A). Furthermore, when EACA, an analog of lysine, was added, the binding of rTpiA to plasminogen was inhibited in a dose‐dependent manner (Fig. 3B).

### rTpiA promotes plasminogen activation

**Fig. 4:**
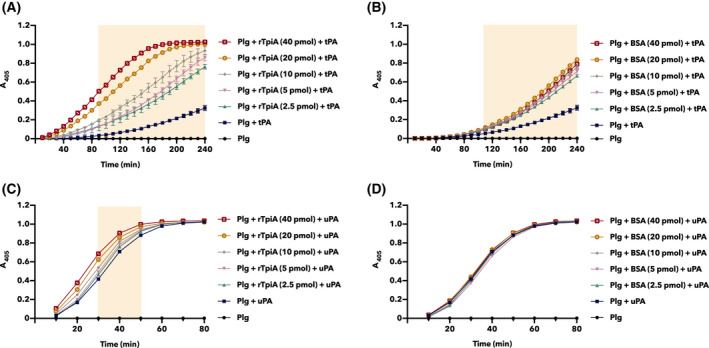
rTpiA promotes plasminogen activation. Plasminogen (2 µg) was preincubated with 2.5 to 40 pmol of rTpiA (A, C) or BSA (B, D), and then 100 ng of tPA (A, B) or uPA (C, D) and the chromogenic substrate S‐2251 were added. The mixtures were incubated at 37 °C, and A_405_ was measured every 10 min. Filled background indicates areas that were significantly higher when preincubated with rTpiA or BSA (at any dose) compared to Plg + tPA or Plg + uPA. Error bars indicate standard error (*n* = 3).

To investigate the effect of the binding of rTpiA to plasminogen on the activation of plasminogen by activators, tPA and uPA, quantitative analysis using chromogenic substrates was performed. Plasminogen was activated by tPA and uPA, and the absorbance of the solution increased in a time‐dependent manner as the plasmin substrate was degraded (Fig. 4). Preincubation of plasminogen with rTpiA or BSA significantly promoted the activation of plasminogen by tPA in a dose‐dependent manner (Fig. 4A,B). However, we confirmed that the promotion of plasminogen activation by tPA was significantly higher in rTpiA than in BSA. By contrast, plasminogen activation by uPA was significantly promoted when plasminogen was preincubated with rTpiA, but not with BSA (Fig. 4C,D). These results indicate that rTpiA not only binds to plasminogen, but also promotes the activation of plasminogen to plasmin.

### rTpiA is degraded by plasmin

**Fig. 5:**
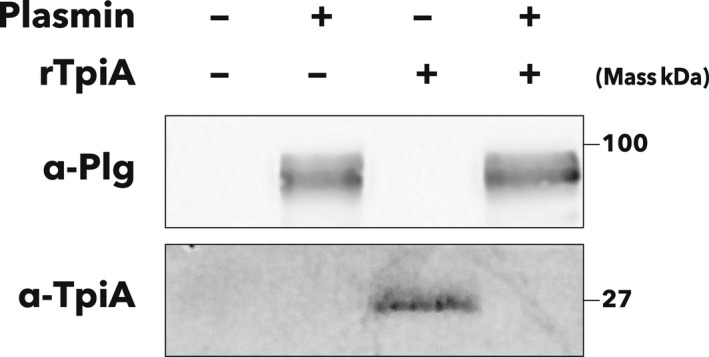
Degradation of rTpiA by plasmin. Plasmin (2 µg), rTpiA (2 µg), and their mixtures (2 µg each) were incubated in PBS at 37 °C for 3 h. They were subjected to SDS/PAGE and electroblotted onto PVDF membranes. The membranes were incubated with antiplasminogen primary antibody (α‐Plg, 1 : 5000 dilution) or anti‐TpiA peptide antibody (α‐TpiA, 1 : 500 dilution), and then incubated with HRP‐conjugated secondary antibody (1 : 3000 dilution). Detection of chemiluminescence due to enzymatic activity of HRP was performed with an exposure time of less than 10 s. This experiment was performed at least thrice, and the same results were obtained.

It has been reported that TPI of *S. aureus* is degraded by plasmin [11]. Therefore, the interaction between rTpiA and plasmin was investigated. After incubation of rTpiA with plasmin, each protein in the mixture was detected by western blotting (Fig. 5). In plasmin alone and rTpiA alone, each protein was detected by antibodies specific for each protein, but no band of rTpiA was detected in their mixtures. Thus, consistent with the report on TPI of *S. aureus*, it was suggested that rTpiA is degraded by the proteolytic activity of plasmin.

### LytA‐mediated cell lysis greatly affects the extracellular release of TpiA in *S. pneumoniae*

**Fig. 6:**
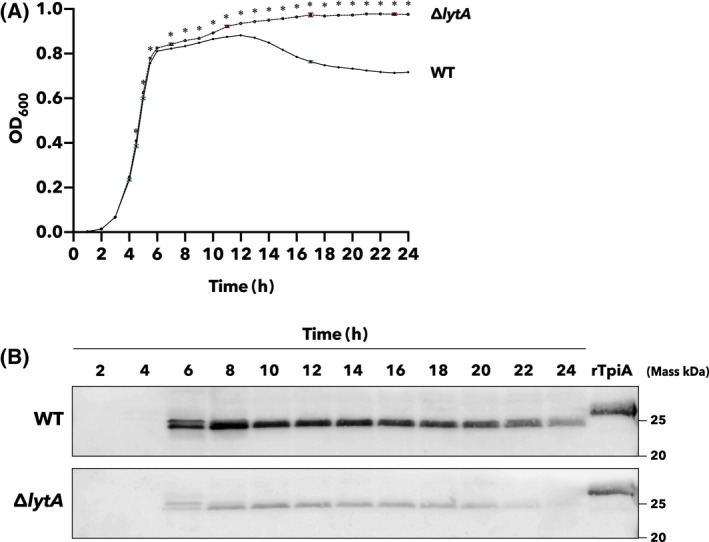
Secretion of TpiA into culture supernatant is dependent on LytA in *S*. *pneumoniae*. (A) Growth curves of wild‐type (WT) and Δ*lytA* strains of *S*. *pneumoniae* D39. Strains were cultured in THY medium, and OD_600_ was measured over time. Asterisks indicate significant differences between groups (*P* < 0.01, Sidak’s multiple comparisons test). Error bars indicate standard error (*n* = 6). (B) Detection of TpiA in culture supernatant. Wild‐type and Δ*lytA* strains of *S*. *pneumoniae* D39 were cultured in THY medium, and culture supernatants were collected over time, concentrated 10‐fold by TCA precipitation, and subjected to SDS/PAGE; protein bands were electroblotted onto PVDF membranes. The membranes were incubated with anti‐TpiA peptide antibody (1 : 500 dilution) and then with HRP‐conjugated secondary antibody (1 : 3000 dilution). rTpiA (200 ng) was used as control. Detection of chemiluminescence due to enzymatic activity of HRP was performed with an exposure time of 5 s. These experiments were conducted at least thrice, and same results were obtained.

Finally, we analyzed the mechanism by which TpiA is released from pneumococcal cells. Since TpiA is an enzyme in the glycolytic system that functions only inside the bacterial cell, it has no signal sequence for extracellular secretion. We hypothesized that LytA‐induced cell lysis is the most influential factor in the extracellular release of TpiA. This is because *S. pneumoniae* is a unique bacterium that autolyzes with the enzyme LytA [26]. The Δ*lytA* mutant strain of *S. pneumoniae* D39 was constructed to elucidate the relationship between LytA and TpiA release. Comparing the growth of the Δ*lytA* mutant strain with that of the wild‐type strain, the turbidity of the Δ*lytA* mutant strain was higher than that of the wild‐type strain in the middle of the exponential growth phase (Fig. 6A). The turbidity of the culture medium of the wild‐type strain decreased after 12 h, but that of the Δ*lytA* mutant did not. These results suggest that autolysis occurred in the wild‐type strain. The culture supernatants of both strains were then collected over time, and TpiA in the supernatants was detected by western blotting (Fig. 6B). Peptide antibodies detected bands of TpiA in the culture supernatant of both wild‐type and Δ*lytA* mutant strains from 6 h after incubation. We quantified the bands of TpiA in both strains using the Fiji image processing package (a variant of ImageJ) [27] and found that the mean of the bands after 6–24 h of incubation of the Δ*lytA* mutant strain was significantly lower than that of the wild‐type strain (Mann‐Whitney test, *P* < 0.0001). This result suggests that a large amount of TpiA is released into the culture supernatant by LytA‐induced bacterial cell lysis.

## Discussion

Proteins that have alternative functions in addition to their main function are called moonlighting proteins [28]. In general, the standard functions of moonlighting proteins are essential for bacterial cell survival and include glycolysis, acting as chaperone proteins, and protein synthesis, and their secondary functions include binding to host epithelial cells, phagocytes, and circulating proteins such as plasminogen [29]. In *S. pneumoniae*, several proteins, including moonlighting proteins, have been reported to bind to host plasminogen: α‐enolase [4, 5, 6, 7, 8, 9, 10], glyceraldehyde‐3‐phosphate dehydrogenase (GAPDH) [30], choline‐binding protein E [31], plasminogen‐ and fibronectin‐binding protein B [32], endopeptidase O (PepO) [33], elongation factor Tu (Tuf) [34], phosphoglycerate kinase [35], and PspC [36].

In the present study, we performed proteomic analysis of the BALF collected from a murine pneumococcal pneumonia model using iTRAQ, and identified 15 pneumococcal proteins (Table 2). *S. pneumoniae* has genes for nearly 2000 different proteins; however only 15 proteins were identified from BALF. Since most of them were housekeeping proteins, which should be expressed under all conditions. These included α‐enolase (phosphopyruvate hydratase), GAPDH, and Tuf, which have been previously reported to contribute to the virulence of *S. pneumoniae*. Among the 15 identified proteins, we focused on TPI, which has been reported to be involved in virulence in other bacterial species. TPI functions as an adhesion factor in *Trichomonas vaginalis* [22], *Lactobacillus plantarum* [37], *Paracoccidiodes brasiliensis* [38], and *S. aureus* [11], interacting with epithelial cells and host proteins such as laminin, fibronectin, and plasminogen [12]. In this study, we analyzed the secondary functions of TpiA, the TPI of *S. pneumoniae*, in addition to its function as an enzyme in the glycolytic system.

The binding of rTpiA to various host proteins was analyzed by far‐western blotting, SPR analysis, and ELISA. rTpiA was shown to have affinity for human plasminogen, indicating that it functions as a binding factor for host proteins (Figs. 1, 2, and 3A). Furthermore, in the presence of EACA, a lysine analog, the binding of rTpiA to plasminogen was inhibited in a dose‐dependent manner (Fig. 3B). This suggests that the binding of rTpiA to plasminogen is dependent on lysine residues in rTpiA. By contrast, rTpiA showed no affinity for fibronectin, for which the TPI of *T. vaginalis* shows binding properties [22], or for elastin, a high‐content ECM of lung tissue [39] (Fig. 3A and Fig. S2). *S. pneumoniae* strains have been reported to have binding properties to laminin, collagen, and plasminogen [40]. There is often more than one binding factor for a given target, as many plasminogen‐binding factors have been reported in *S. pneumoniae*. As Figs. 1, 2, and 3A do not clearly show the affinity of rTpiA for laminin, it is possible that TpiA is not a major binding factor to laminin.

rTpiA significantly enhanced plasminogen activation by tPA and uPA in a dose‐dependent manner (Fig. 4). Here, BSA significantly accelerated the activation of plasminogen by tPA. However, as BSA is used as a stabilizer for restriction enzyme reactions, stabilizing the enzymatic reaction may be a factor in the promoted activation of plasminogen. The activation of plasminogen by tPA was relatively lower than that by uPA; therefore, the reaction stabilizing effect of BSA on promoting plasminogen activation may have been more apparent. As the effect of uPA on plasminogen activation was relatively high, the effect of BSA may not be well reflected. In contrast, in the presence of rTpiA, plasminogen activation by uPA was significantly enhanced. We also confirmed that rTpiA significantly promoted plasminogen activation by tPA compared to BSA. Therefore, even if the stabilizing effect of the addition of other proteins such as BSA is subtracted, the plasminogen activation‐promoting activity of rTpiA is remarkable, and it is obvious that rTpiA has promoting activity. In a previous report, TPI of *S. aureus* inhibited plasminogen activation [11]. The rTpiA of *S. pneumoniae* discussed in this study has the opposite property to this report, and it was found that the secondary function of TPI differs depending on the species. In addition, consistent with a previous report [11], rTpiA was found to be degraded by the proteolytic activity of plasmin (Fig. 5).

The sequences of the homologs of TPI were compared among several related species of *S. pneumoniae*, *S*. *aureus*, *Escherichia coli*, humans and mice (Fig. S3). When compared with the sequence of *S. pneumoniae*, most *Streptococcus* species showed high identity (83.2–99.2%), and *S*. *aureus* showed 58.1% identity (Table S1). The position of the lysine residue of TPI, which is considered to be involved in plasminogen binding, was also different between *S. pneumoniae* and *S. aureus*. These differences in sequence may be responsible for the differences in the function of TPI between the two species, particularly in its interaction with plasminogens.

Moonlighting proteins perform secondary functions in places that differ from their original function. In other words, there are many cases in which proteins that are originally localized inside the cell have other functions outside the cell or on the cell surface [29, 41]. However, they do not contain any known sequence motifs for secretion or anchorage to the bacterial surface layer and how they are released into the extracellular space is unclear. TpiA of *S. pneumoniae* also does not contain secretory or anchoring signals, such as the LPxTG motif [42, 43]. However, *S. pneumoniae* has the unique property of autolysis when it reaches the stationary phase of growth, which releases pneumolysin and other virulence factors from the bacterial cell [26, 44, 45]. Thus, in *S. pneumoniae*, extracellular release of intracellular proteins is readily achieved by autolysis. Fig. 6 shows that TpiA is also released into the culture supernatant in a LytA‐dependent manner, which induces autolysis. In Fig. 6, the molecular weight of TpiA released from the cell is slightly smaller than that of rTpiA, but this is due to the slightly larger size of rTpiA to which His‐tag and signal sequences are fused (calculated molecular weights are approximately 28 000 for rTpiA and approximately 26 600 for ‘natural’ TpiA). Furthermore, TpiA was detected in the extracted fraction (extraction method with 8M urea [46, 47, 48]) of *S. pneumoniae* cell‐surface proteins (Fig. S4). This signal was more evident in the wild‐type strain than in the Δ*lytA* mutant strain. This suggests that TpiA released from the cells by autolysis is present not only in the culture medium but also on the cell surface of *S. pneumoniae*.

The surface of the pulmonary epithelium faces the external environment and is the first barrier to the entry of *S. pneumoniae* infecting the lungs into the host tissue. BALF contains a fibrinolytic system from which *S. pneumoniae* can recruit plasminogen [31, 49]. Far‐western blotting of BALF samples from a pneumococcus‐infected mouse with plasminogen and detection of plasminogen‐bound bands revealed a band of size corresponding to TpiA (Fig. S5). This result suggests that TpiA can bind specifically to plasminogen in BALF. This is supported by the fact that rTpiA significantly binds to plasminogens among various host proteins (Fig. 3A). Plasminogen bound to the bacterial cell surface is activated to plasmin, which acquires proteolytic activity, degrades ECM proteins, and facilitates bacterial migration on the human ECM [2, 50]. In addition, active plasmin disrupts intercellular junctions and facilitates the passage of the cell barrier [51]. The present study suggests that pneumococcal TpiA facilitates these processes.

In conclusion, TpiA of *S. pneumoniae* functions intracellularly as an enzyme in the glycolytic system, but is released extracellularly by LytA‐dependent autolysis. The released TpiA binds to host plasminogen and promotes plasmin activation, that is, conversion to plasmin, suggesting that it contributes to entry into host tissues. The identification of TpiA as a novel virulence factor was the result of the proteomic study, which can be used to capture the actual phenomena occurring *in vivo* and is considered to be an effective approach.

## Conflict of interest

The authors declare no conflict of interest.

## Author contributions

SH, HD, and YT conceived and supervised the research; SH and HD designed the experiments; SH, HD, TH and TI performed the experiments; TI, HT, KS, and FM contributed to the analysis and interpretation of the experimental data; SH wrote the manuscript; HD and YT revised the manuscript.

## Supporting information


**Fig. S1.** Original images of the biding assay of rTpiA to host proteins.
**Fig. S2.** Binding assay of rTpiA to fibronectin and elastin.
**Fig. S3.** Amino acid sequence alignment of TPI for each species.
**Fig. S4.** Detection of TpiA in the surface protein fractions of wild‐type and the ΔlytA mutant strains of *S. pneumoniae* D39.
**Fig. S5.** Binding assay of plasminogen to BALF samples from murine pneumococcal pneumonia model by far‐western blotting.
**Table S1.** Homology of the amino acid sequence of TPI to *S. pneumoniae* D39.

## Data Availability

Data supporting the results of this study are included within this paper and in the Supplemental File. These data are not archived in any public repository.
