# Supplementary material for: Triosephosphate isomerase of Streptococcus pneumoniae is released extracellularly by autolysis and binds to host plasminogen to promote its activation
Source: FEBS Open Bio. 2022 Mar 29;12(6):1206–19. doi: 10.1002/2211-5463.13396 (PMC9157410; doi:10.1002/2211-5463.13396)
Supplement: Supplementary file 1 — Fig. S1. Original images of the biding assay of rTpiA to host proteins. Fig. S2. Binding assay of rTpiA to fibronectin and elastin. Fig. S3. Amino acid sequence alignment of TPI for each species. Fig. S4. Detection of TpiA in the surface protein fractions of wild‐type and the ΔlytA mutant strains of S. pneumoniae D39. Fig. S5. Binding assay of plasminogen to BALF samples from murine pneumococcal pneumonia model by far‐western blotting. Table S1. Homology of the amino acid sequence of TPI to S. pneumoniae D39. [file FEB4-12-1206-s001.pdf]

Supplementary Figures and Table

Triosephosphate isomerase of *Streptococcus pneumoniae* is released extracellularly by autolysis and binds to host plasminogen to promote its activation

Satoru HIRAYAMA [1], Hisanori DOMON [1,2], Takumi HIYOSHI [1,2,3], Toshihito ISONO [1], Hikaru TAMURA [1,3], Karin SASAGAWA [1,3], Fumio TAKIZAWA [1,3] and Yutaka TERAO [1,2]

[1] *Division of Microbiology and Infectious Diseases, Niigata University Graduate School of Medical and Dental Sciences, Niigata, Japan*
[2] *Center for Advanced Oral Science, Niigata University Graduate School of Medical and Dental Sciences, Niigata, Japan*
[3] *Division of Periodontology, Niigata University Graduate School of Medical and Dental Sciences, Niigata, Japan*

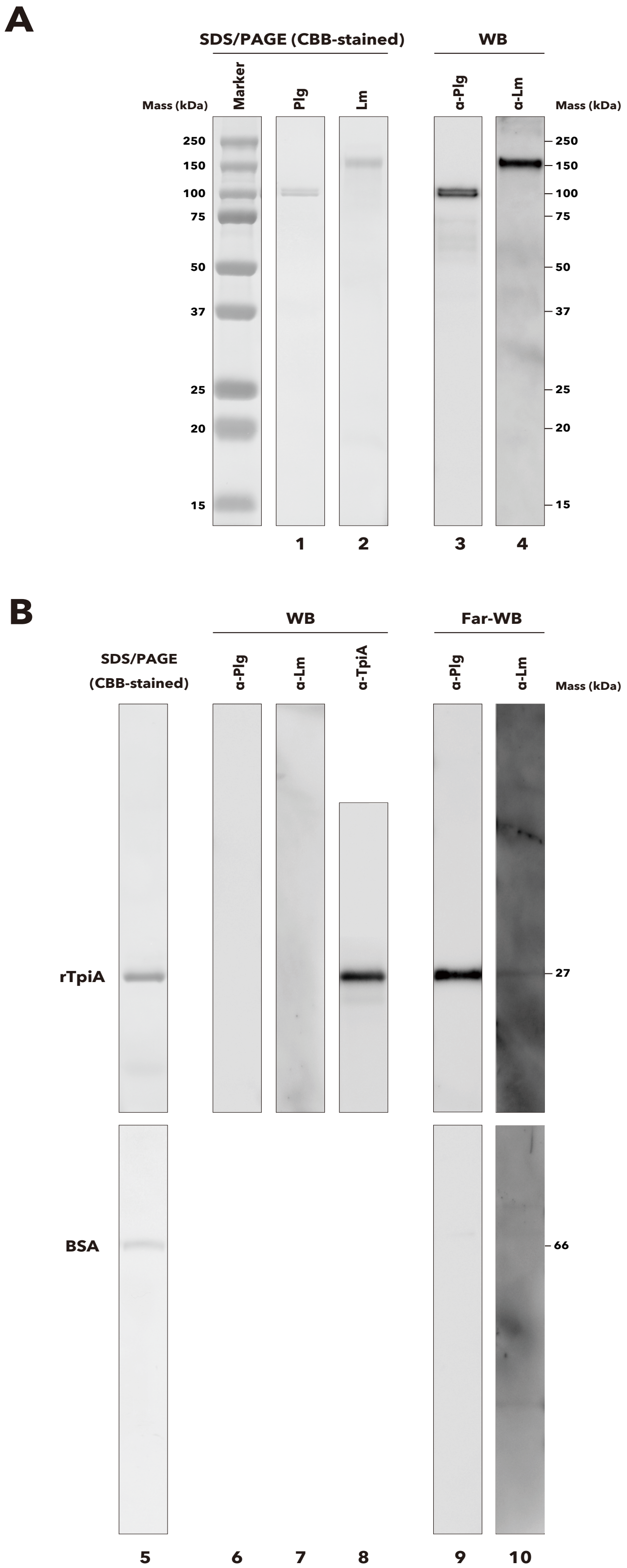

Supplementary Fig. S1 Original images of the biding assay of rTpiA to host proteins.

Unprocessed images of the images shown in Fig. 1.

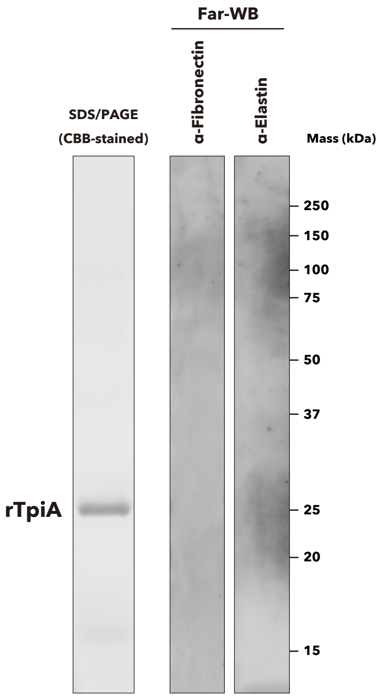

Supplementary Fig. S2 Binding assay of rTpiA to fibronectin and elastin.

Binding assay of rTpiA to fibronectin or elastin using far-western blotting. rTpiA (200 ng) was subjected to SDS/PAGE, and the proteins were electroblotted onto PVDF membranes. The membranes were incubated with 30 µg/mL fibronectin or elastin, followed by incubation with anti-fibronectin (1:1000 dilution, Sigma-Aldrich) or anti-elastin (1:1000 dilution, GeneTex) primary antibodies, followed by incubation with HRP-conjugated secondary antibodies (1:3000 dilution). SDS/PAGE and CBB staining were performed to detect rTpiA. Chemiluminescence due to the enzymatic activity of HRP was detected with an exposure time of 2 s (fibronectin) or 30 s (elastin). These experiments were conducted at least thrice, and the same results were obtained.

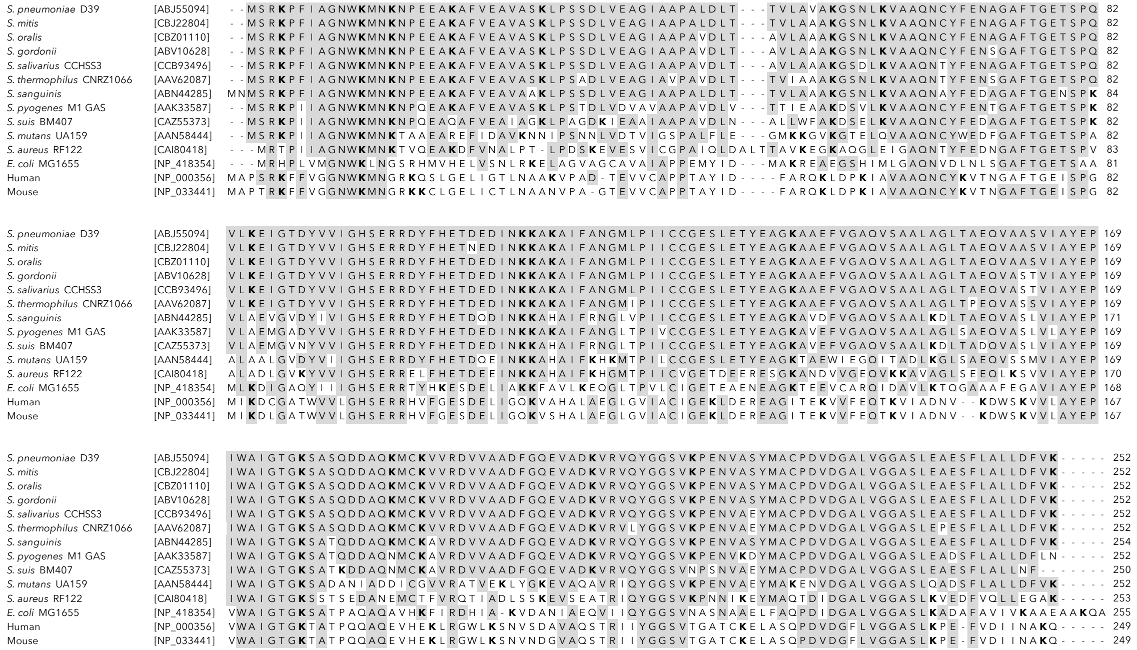

Supplementary Fig. S3 Amino acid sequence alignment of TPI for each species.

The alphanumeric characters in parentheses indicate the accession numbers. The highlighted sequences were consistent with those of *S. pneumoniae* D39. Lysine residues are indicated in bold.

Supplementary Table S1. Homology of the amino acid sequence of TPI to *S. pneumoniae* D39.

	Accession number	Identity [%]	Similarity [%]	Gap [%]
Streptococcus mitis	CBJ22804	99.2	99.6	0.0
Streptococcus oralis	CBZ01110	98.8	99.2	0.0
Streptococcus gordonii	ABV10628	97.6	99.2	0.0
Streptococcus salivarius CCHSS3	CCB93496	96.8	98.4	0.0
Streptococcus thermophilus CNRZ1066	AAV62087	94.8	97.2	0.0
Streptococcus sanguinis	ABN44285	90.9	95.2	0.0
Streptococcus pyogenes M1 GAS	AAK33587	86.1	92.4	0.0
Streptococcus suis BM407	CAZ55373	83.2	90.0	0.0
Streptococcus mutans UA159	AAN58444	66.3	80.2	0.0
Staphylococcus aureus RF122	CAI80418	58.1	71.9	1.6
Escherichia coli MG1655	NP_418354	42.7	58.5	0.4
Human	NP_000356	39.7	54.6	2.5
Mouse	NP_033441	39.2	53.9	4.9

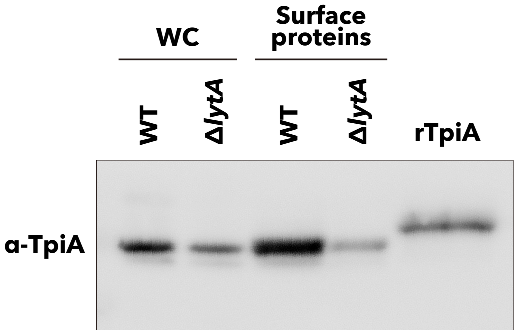

Supplementary Fig. S4 Detection of TpiA in the surface protein fractions of wild-type and the Δ*lytA* mutant strains of *S. pneumoniae* D39.

Wild-type (WT) and Δ*lytA* mutant strains of *S. pneumoniae* D39 were cultured in THY medium for 8 h, and whole-cell (WC) samples and cell surface protein fractions were collected. Cell surface proteins were extracted by 8 M urea. Briefly, bacterial cells were collected from 35 mL of the culture and washed with PBS. They were then suspended in 1 mL of 8 M urea and stirred for 1 h at room temperature, and the supernatant obtained by centrifugation was used as the surface protein fraction. WC samples (0.06 OD unit), surface protein fractions (1.25 μL), and rTpiA (200 ng) were subjected to SDS-PAGE, and the proteins were electroblotted onto PVDF membranes. The membranes were incubated with an anti-TpiA peptide antibody (α-TpiA, 1:500 dilution) and then with an HRP-conjugated secondary antibody (1:3000 dilution). Chemiluminescence due to the enzymatic activity of HRP was detected with an exposure time of 4 s. These experiments were conducted at least thrice, and the same results were obtained.

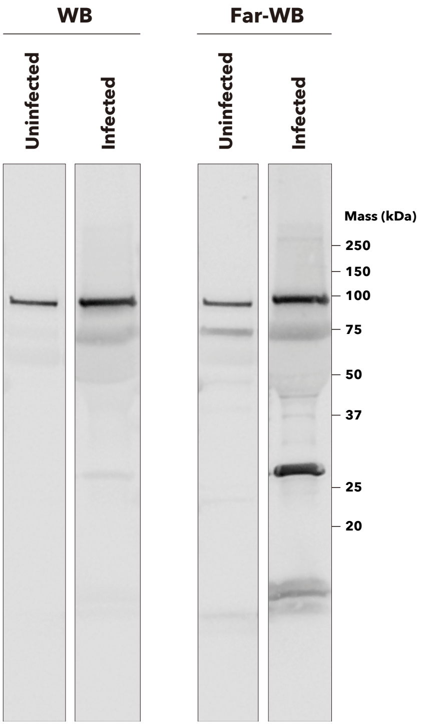

Supplementary Fig. S5 Binding assay of plasminogen to BALF samples from murine pneumococcal pneumonia model by far-western blotting.

BALF samples (18 µL) were subjected to SDS-PAGE, and the proteins were electroblotted onto PVDF membranes. The membranes were incubated with (for far-western blotting) or without (for western blotting) 30 µg/mL of plasminogen, followed by incubation with anti-plasminogen (1:5000 dilution) primary antibody and HRP-conjugated secondary antibody (1:3000 dilution). Chemiluminescence due to the enzymatic activity of HRP was detected with an exposure time of 2 s. These experiments were conducted at least thrice, and the same results were obtained.
